# Complete Response in Hairy Cell Leukemia to Anti-CD22 CAR T-Cell Therapy

**DOI:** 10.1200/PO-25-00269

**Published:** 2025-07-16

**Authors:** Robert J. Kreitman, Brett Schroeder, Evgeny Arons, Constance Yuan, Hao-Wei Wang, Mark Raffeld, Liqiang Xi, Stefania Pittaluga, Hong Zhou, Mory Gould, Isaac Shpilman, Evrim Turkbey, Katherine R. Calvo, Lacey James, Olena Sierra Ortiz, Steve Highfill, David Stroncek, Bonnie Yates, Ira Pastan, Nirali N. Shah

**Affiliations:** ^1^ Laboratory of Molecular Biology, National Cancer Institute, NIH, Bethesda, MD; ^2^ Medical Oncology Branch, National Cancer Institute, NIH, Bethesda, MD; ^3^ Laboratory of Pathology, National Cancer Institute, NIH, Bethesda, MD; ^4^ Radiology and Imaging Sciences, Clinical Center, NIH, Bethesda, MD; ^5^ Department of Laboratory Medicine, Clinical Center, NIH, Bethesda, MD; ^6^ Office of Research Nursing, National Cancer Institute, NIH, Bethesda, MD; ^7^ Department of Transfusion Medicine, Clinical Center, NIH, Bethesda, MD; ^8^ Pediatric Oncology Branch, National Cancer Institute, NIH, Bethesda, MD

## Introduction

Classic hairy cell leukemia (HCL) is a rare B-cell malignancy expressing CD11c, CD22, CD25, CD103, CD123, annexin A1 (Anxa1), and *BRAF* V600E mutation.^1,2^ Purine analogs cladribine and pentostatin achieve complete remissions (CR), but relapse occurs at a median 4.5-16 years depending on age and whether follow-up included bone marrow or just blood counts.^3,4^ Retreatment is effective but with cumulative toxicities and relapses of decreasing intervals. Nonchemotherapy treatments with reported efficacy in HCL include anti-CD20 monoclonal antibodies rituximab and obinutuzumab, anti-CD22 recombinant immunotoxin moxetumomab pasudotox (Moxe), BRAF/MEK inhibitors, BTK inhibitor ibrutinib, BCL2 inhibitor venetoclax, and allogeneic transplantation.^5-13^ These treatments except for BRAF inhibition may also treat HCL variant (HCLv), which is more refractory and usually lacks CD25, CD123, Anxa1, and *BRAF* V600E.^6,14^ Salvage treatments for HCL/HCLv are usually of temporary benefit. Chimeric antigen receptor T-cell (CAR-T) therapy has been developed as an engineered autologous cell-based therapy useful in B-cell malignancies^15^ but has not yet been reported in HCL/HCLv.

To target CAR-T cells to CD22+ malignancies, the MAb m971 was used, which binds domains 5-7 of CD22 more proximal to the cell membrane than would Moxe.^5,16^ The CAR construct incorporates second-generation lentiviral vector pLTG1418 with the m971BBZ construct under an EF1α promoter integrating self-inactivating sites. In a phase I trial in predominantly B-ALL, anti-CD22 CAR-T cells achieved a CR rate of 70% in 58 patients with a median relapse-free survival of 6 months and an overall survival of 13.4 months.^17^ Minimal residual disease (MRD)–negative CR correlated with higher CD22 site density. In most relapses, CD22 was negative/dim, indicating selection for lower CD22 expression (antigen escape). Outcomes for CD22 CAR-T cells in adult relapsed/refractory large B-cell lymphoma have also been favorable.^18^ Since CD22 density is much higher in HCL/HCLv compared with B-ALL (median 44,000 *v* 3,500 sites/cell), and since Moxe achieved high rates of MRD-negative CR without selection for CD22 dim/negative HCL cells, we tested anti-CD22 CAR-T in HCL/HCLv to evaluate toxicity and determined whether responses would be deep and durable without CD22 antigen escape.

## Methods

The study (ClinicalTrials.gov identifier: NCT04815356) was performed in accordance with the Declaration of Helsinki. All patients signed an informed consent document approved by the National Cancer Institute Investigator's Review Board. Eligibility required relapsed/refractory HCL/HCLv after previous purine analog and BRAF inhibition (if BRAF V600E+) and need for treatment due to cytopenias or symptomatic HCL/HCLv masses (protocol). Patients received lymphodepleting chemotherapy with fludarabine 30 mg/m^2^ days –5, –4, –3, and –2 and cyclophosphamide 500 mg/m^2^ days –3 and –2 (fludarabine-cyclophosphamide [FC]), followed by CAR-T infusion day 0.

## Results

### Early Cases at Dose Level 1

Patients 1 and 2 receiving 1 × 10^5^ CAR-T cells/kg (Table 1) had inadequate engraftment and no significant toxicity or response. The trial was subsequently amended to enroll patients at 3 × 10^5^ CAR-T cells/kg, the active dose in the B-ALL trial.^17^

**TABLE 1.** Baseline Clinical Data and Mutations Found in Patients With HCL/HCLv Treated With CAR-T

Patient No.	Patient 1	Patient 2	Patient 3	Patient 4
Diagnosis	HCL	HCLv	HCL	HCL
Age	57	65	70	68
Sex	Male	Male	Male	Male
Diagnosis to CAR-T	10.7 years	15.7 years	33.0 years	38.1 years
Previous treatment	CDA+R, R, CDA, Vemu, DCF, Moxe-Vemu-R, Splenectomy, Enco-Bini, DCF+R	CDA+R, Splenectomy, R, DCFR, BR, Ibrutinib, Bini	CDA, R, DCF, R, Dabr-Tram, MoxeR, DCF	Splenectomy, IFN, DCF, CDA, DCFR, VemuR, Vemu
Neutrophils on day 0	2.27 cells/nL	5.66 cells/nL	1.503 cells/nL	8.744 cell/nL
Hemoglobin on day 0	7.9 g/dL	11.7 g/dL	7.1 g/dL	13 g/dL
Platelets on day 0	106 cells/nL	373 cells/nL	14 cells/nL	215 cells/nL
HCL count Pre–CAR-T	647 cells/mm^3^	0 cells/mm^3^	56,242 cells/mm^3^	0 cells/mm^3^
BMBx	85% HCL	0% HCLv	80%-90% HCL	0% HCL
Involvement	Lymph nodes	Lymph nodes	Splenomegaly	Chest wall, bladder
Mutations	BRAF V600E (44%) ATM R2032K (93%) KRAS G12D (3%) NRAS G12S (7%) ARID1A Q67* (62%)	TP53 V272M (2%) CDKN2A G35A (47%)	BRAF V600E (61%) EZH2 C668fs*9 (4%) SMARCA4 E670* (23%) DNMT3A E784* (3%) MUTYH, Q110* (5%)	BRAF V600E (49%) BCOR N1459S (70%) SF3B1 R625H (36%) SF3B1 R625C (11%)
CAR-T cell dose	10^5^ cells/kg	10^5^ cells/kg	3 × 10^5^ cells/kg	3 × 10^5^ cells/kg
Response to CAR-T	No response	No response	>5-log HCL reduction	MRD-free CR

NOTE. The Involvement row lists measurable sites of HCL/HCLv outside the bone marrow, which also constituted indications for treatment. Mutations were determined by TSO500 NGS of 523 genes. VAF are listed in parentheses after each mutation. The number of doses per course was usually 5-7 for cladribine (CDA), 6-12 for pentostatin (DCF), and 4-8 for rituximab (R).

Abbreviations: BMBx, bone marrow biopsy % infiltration; CAR-T, chimeric antigen receptor T cell; CDA, 2-chlorodeoxyadenosin or cladribine; CDAR, CDA plus rituximab (R) given concurrently; CDA+R, CDA followed by delayed R; CR, complete remissions; Dabr-Tram, dabrafenib-trametinib; DCF, deoxycoformycin or pentostatin; DCFR, DCF + R, Enco-Bini, encorafenib and binimetinib; HCL, hairy cell leukemia; HCLv, HCL variant; IFN, interferon; Moxe, moxetumomab pasudotox; MRD, minimal residual disease; NGS, next generation sequencing; VAF, variant allele frequencies; Vemu, Vemurafenib.

### Dose Level 2

Patient 3 was a 70-year-old male with a 33-year history of HCL, multiply pretreated (Table 1) with transfusion-dependent pancytopenia. Circulating HCL count was 56,242/mm^3^. Following lymphodepletion and CAR-T infusion at 3 × 10^5^ CAR-T cells/kg, he developed cytokine release syndrome (CRS, max grade 1) on day +12, immune effector cell–associated neurotoxicity syndrome (max grade 2) on day +26, and immune effector cell–associated hemophagocytic lymphohistiocytosis-like syndrome (IEC-HS; max grade 4) on day +19. IEC-HS presented with increased ferritin from baseline 1,687 to 196,000 by day +27. IEC-HS–directed therapy included steroids, anakinra, siltuximab, emapalumab, and etoposide, resulting in marked improvement with normalizing inflammatory markers. CAR-T cells peaked at 34% of T cells on day +27. Importantly, HCL count decreased from 28,000/mm^3^ on day 0 to <0.2/mm^3^ by day +30, but transfusion-dependent pancytopenia persisted. Unfortunately, on day +32, before resolution of HCL-related cytopenias could be expected, the patient developed septic shock with neutropenic colitis. Autopsy showed no evidence for active hemophagocytosis and revealed residual HCL in marrow and spleen.

### Index Case

**FIG 1.**
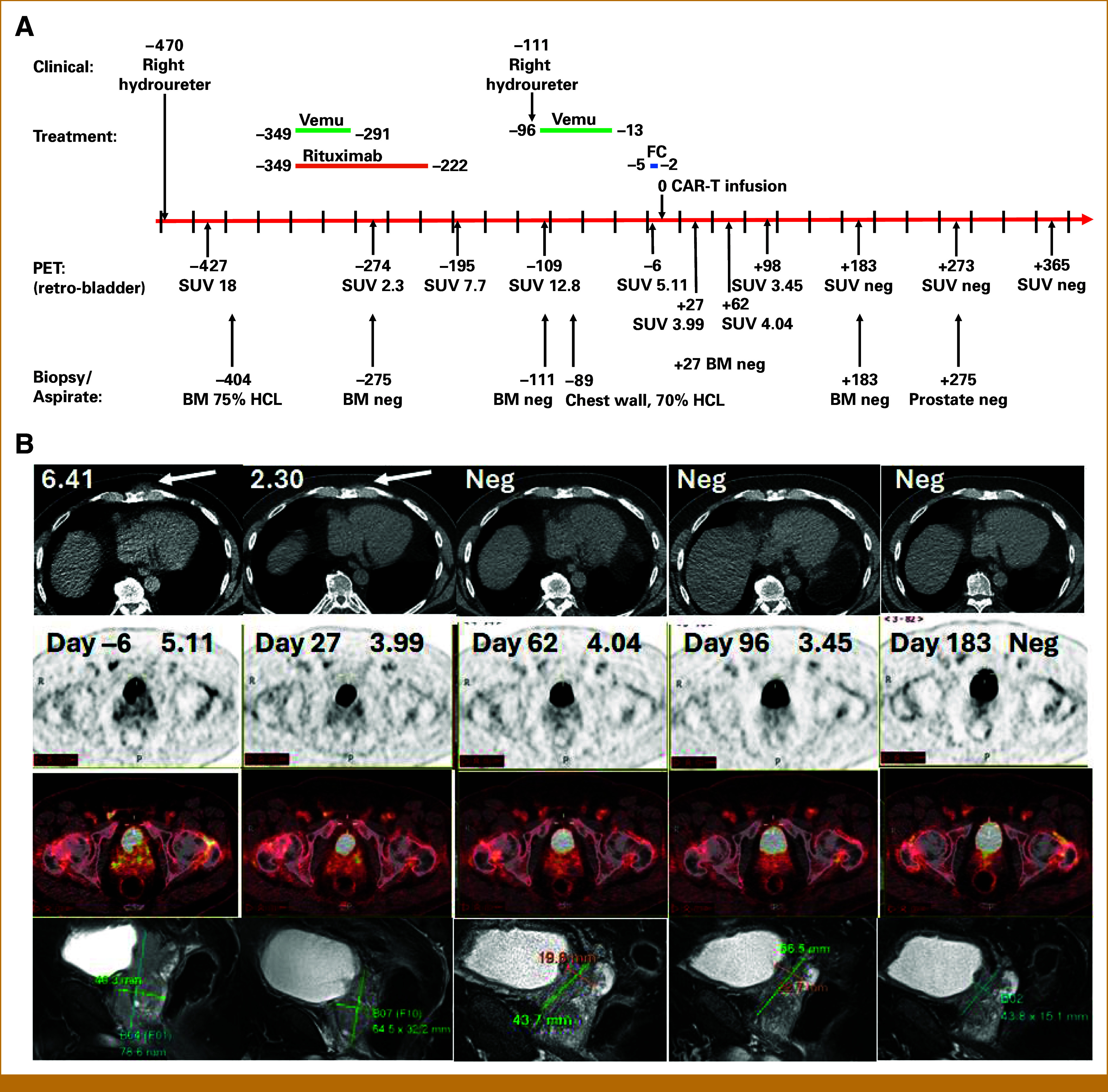
Timeline of treatments and response. (A) Timeline from top to bottom shows clinical presentation of right hydroureter due to posterior bladder (prostate) mass, treatments, PET SUV of posterior (retro-) bladder mass, and biopsy/aspirate of BM, chest wall mass, and posterior bladder (prostate) area. All events include day numbers before (negative) and after (positive) CAR-T infusion on day 0. Start and stop days of Vemu, rituximab, and FC lymphodepleting chemotherapy are shown. Not shown are results of blood flow cytometry to quantify HCL cells, sensitive to 0.002%, which was positive on day +7 but negative on days –160 (when first evaluated at NIH), –111, –69, –34, –6, +14, +20, +27, +62, +97, +183, +273, and +365. (B) PET scan images are shown in the top three rows with day numbers and SUVs. The bottom row shows pelvic MRIs at the same time points as the PET images. Images on days +273 and +365 are not shown but unchanged from +183. BM, bone marrow; CAR-T, chimeric antigen receptor T cell; FC, fludarabine-cyclophosphamide; HCL, hairy cell leukemia; MRI, magnetic resonance imaging; NIH, National Institutes of Health; PET, positron emission tomography; SUV, standardized uptake value; Vemu, vemurafenib.

Patient 4 is a 68-year-old male with a 38-year history of HCL, multiply pretreated (Table 1). Approximately 15 months before CAR-T infusion, he presented with right hydroureter due to a posterior bladder mass near the prostate (Fig 1A), with maximum standardized uptake value (SUV) of 18 on positron emission tomography (PET) scan. Other areas of disease by PET included an anterior chest wall mass (SUV 9), a cardiophrenic mass (SUV 6.3), and a hepatic mass (SUV 8.6). Chest wall mass biopsy confirmed BRAF V600E–positive HCL. Concurrent bone marrow showed 75% HCL infiltration. He subsequently initiated treatment with vemurafenib and rituximab, with response lasting only 5 months, followed by progression <1 month after finishing rituximab, as shown in Figure 1A.

**FIG 2.**
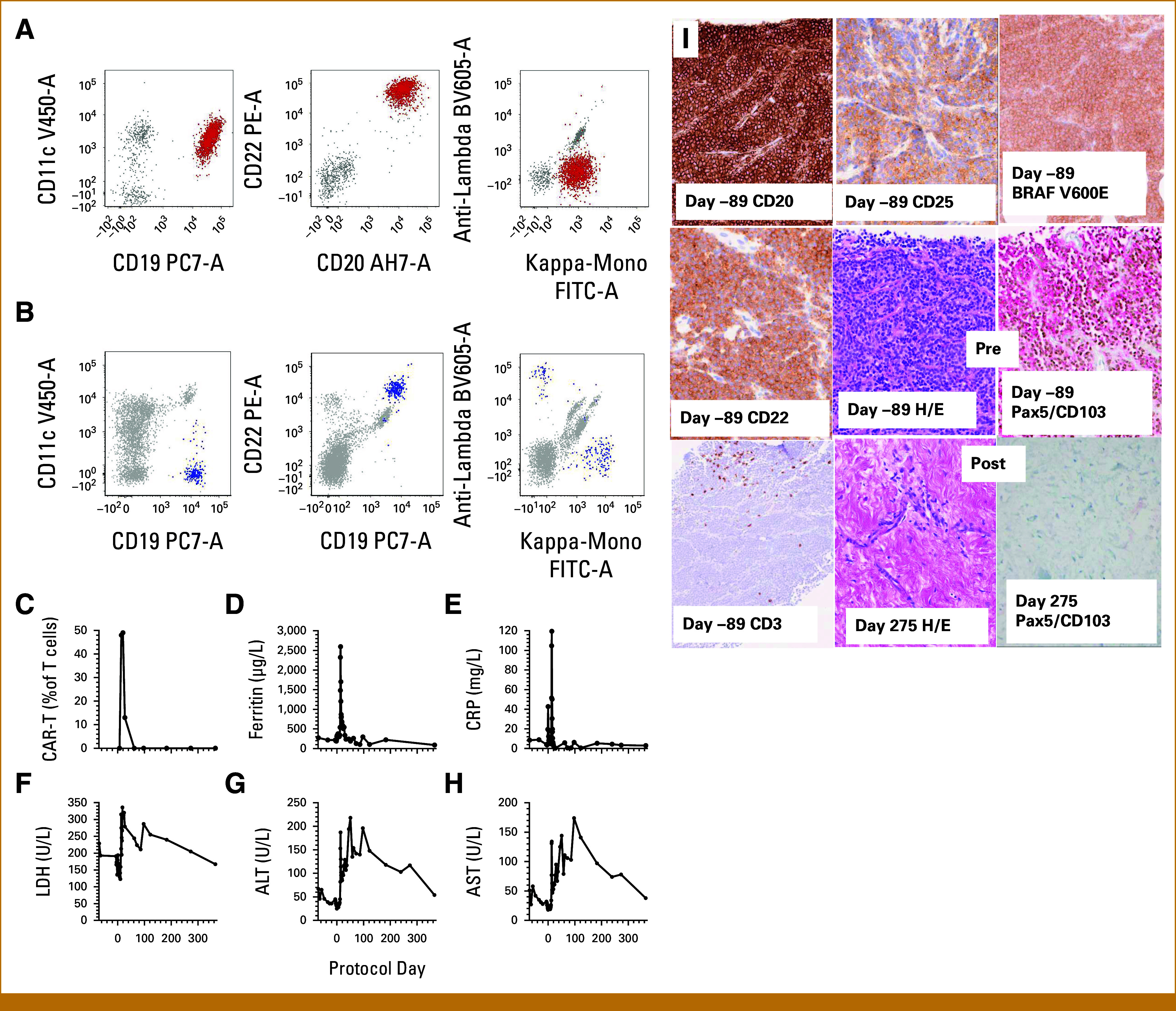
Characterization of HCL and lab abnormalities in patient 4. (A) Flow cytometry of an FNA sample of the chest wall mass from day –110 revealed a prominent population of monoclonal B cells (gated/colored red) expressing CD19, bright CD20, bright CD25 (5,286 sites/cell), CD103, CD11c, bright CD22 (47,540 sites/cell) and restricted expression of kappa surface light chain immunoglobulin. (B) FNA flow cytometry of the posterior bladder (prostate) mass on day +275, gating on mononuclear cells, showed no evidence of HCL, with polyclonal B cells highlighted in blue. Circulating CAR-T cells, detected by flow cytometry at a threshold of >1% of T cells, were 48% by day +14, 49% by day +20, 13% by day +27, and were undetectable starting day +62 (C). Graphs also show ferritin (D), CRP (E), LDH (F), ALT (G), and AST (H) from days –67 to +365. (I) Core biopsy of the chest wall mass from day –89 showed an HCL infiltrate with IHC positive in nearly all HCL cells for CD20, CD25, BRAF V600E, CD22, H/E, and Pax5/CD103 double stain, but not CD3. The two images in the lower right show the H/E and Pax5/CD103 stains of the core biopsy of the posterior bladder (prostate) mass on day +275, confirming elimination of all HCL cells. CAR-T, chimeric antigen receptor T cell; CRP, C-reactive protein; FNA, fine needle aspirate; HCL, hairy cell leukemia; LDH, lactate dehydrogenase.

On the basis of recurrent hydroureter and progressive disease, he was referred to National Institutes of Health for enrollment to the CD22 CAR-T cell trial. Bone marrow biopsy and flow cytometry of bone marrow aspirate and blood were negative on day –111 (3.5 months before CAR-T infusion) attributed to recent rituximab (last dose day –222, 7.3 months before CAR-T infusion), but SUVs of the anterior chest wall and posterior bladder masses were 13.6 and 12.8, respectively, on day –109. Following apheresis on day –98 to obtain T cells for engineering CAR-T cells, vemurafenib was resumed on day –96 for bridging therapy to avoid nephrostomy. On day –89 (3 months before CAR-T infusion), core biopsy of the chest wall mass demonstrated ongoing disease, expressing CD20, CD25, BRAF V600E, CD22, and Pax5/CD103 by immunohistochemistry (Fig 2I) and CD25, CD103, CD123, CD20, CD11c, CD19, CD22, and kappa light chain by flow cytometry (Fig 2A). Vemurafenib was stopped on day –13, and on day –6, the SUV of the posterior bladder was still positive at 5.11 (Figs 1A and 1B).

FC lymphodepleting chemotherapy was administered from day –5 to –2, followed by CAR-T infusion on day 0 (Fig 1A). CAR-T infusion was well tolerated. By day +7, 3.4% of circulating B cells were consistent with HCL and CAR-T cells were not yet detectable. Maximum CRS grade 1 began day +11 and responded to a single dose of tocilizumab 8 mg/kg. By day +14, 2 weeks after CAR-T infusion, flow cytometry documented 48% of circulating T cells consistent with CAR-T, and once CAR-T cells appeared, HCL cells became undetectable. Transient elevations in hepatic transaminases which fluctuated through 9 months after infusion were attributed to CAR-T cells targeting HCL cells in the liver (Figs 2G and 2H). Despite transient elevations in inflammatory markers, including ferritin (Fig 2D) with CRS onset, IEC-HS did not develop.

At day +27 (1 month after CAR-T infusion), the SUV of the posterior bladder mass decreased to 3.99 and concurrent bone marrow biopsy and aspirate remained MRD-negative. Repeated evaluations demonstrated clearance of any PET-avid lesion by 6 months after infusion (Fig 1B). Subsequent biopsy (four cores) and flow cytometry of the residual posterior bladder (prostate) mass (Fig 2) and repeat PET scan at 9 months after infusion were negative for HCL, confirming MRD-free CR. PET remained negative at the 1-year time point.

## Discussion

Although CAR-T cells were undetectable after day +27, they possibly persisted at low levels at sites of residual disease such as liver, bone marrow, and the posterior bladder (prostate) mass, continuing to eliminate HCL cells. First appearance of CAR-T cells on day +14 was associated with complete clearing of circulating HCL cells, which remained negative through day +365 (1 year after infusion). This excludes FC (day –5 to –2) as the cause of HCL clearance from blood. HCL cells also remained negative in bone marrow through day +183 (6 months after infusion). It is notable that the previous remission to vemurafenib-rituximab lasted <5 months after that treatment began, with rapid progression noted <1 month after the last dose of rituximab. The aggressiveness of this patient's classic HCL might be related to the BCOR N1459S mutation, present in addition to BRAF V600E as a major subclone (Table 1). The BCOR N1459S mutation was not reported in HCL/HCLv but was documented most often in endometrial tumors and also in B-cell malignancies like chronic lymphocytic and prolymphocytic leukemia.^19,20^

Although CD22 is usually brighter in HCL than CD19, targeting the latter by CAR-T would also be potentially useful. Targeting using CD19/CD22 CAR-T cells, which were already tested in B-ALL,^15^ might provide even more sites/cell.

Collectively, we demonstrate that at 3 × 10^5^/kg, CD22 CAR-T cells can effectively eradicate relapsed/refractory HCL. The difference in toxicity and outcome between patients 3 and 4, who received the same dose of CAR-T cells and had comparable CD22 site density (44,799 and 47,540 sites/cell, respectively), was likely due to the difference in disease burden. Patient 3 had extensive involvement in the bone marrow and blood, and patient 4 was negative in those areas at the time of CAR-T infusion. As seen with B-ALL, higher disease burden associates with heightened toxicity.^17^ In subsequent patients, we plan to debulk HCL/HCLv tumor burden before CAR-T infusion using other HCL treatments to at least transiently minimize circulating HCL. Enrollment on this study continues with this promising anti-CD22 therapy.
